# Comparison and identification of early clinical, biological and echocardiographic prognostic markers in cardiac amyloidosis

**DOI:** 10.1186/1750-1172-10-S1-P60

**Published:** 2015-11-02

**Authors:** Thibaud Damy, Arnaud Jaccard, Aziz Guellich, David Lavergne, François Deux Jean, Jehan Dupuis, Valérie Frenkel, Dania Mohty

**Affiliations:** 1 CHU H Mondor, Amyloidosis Mondor Creteil, 94000, Créteil, France; 2 CHU Limoges, AL Amyloidosis Referral Center, 87000, Limoges, France

## Background

The early prognosis of amyloidosis is known to depend heavily on cardiac function and may be improved by identifying patients at highest risk for adverse cardiac events. We looked for early predictors of mortality in patients with cardiac AL amyloidosis, hereditary transthyretin amyloidosis (m-TTR), or senile transthyretin amyloidosis (WT-TTR).

## Method

Prospective observational study of 198 patients seen at two French university centers.

## Results

NYHA class was III-IV in 31% of patients. Median (25th-75th percentile) values were 69 (60-76) years for age, 3027 (673-7155) pg•mL-1 for NT-proBNP, and 60% (48-66) for left ventricular ejection fraction. Interventricular septal thickness was greater in the m-TTR and WT-TTR groups than in the AL group (P<0.0001). NT-proBNP correlated with IVST (R=0.34; P=0.0001). The 6-month mortality rate was 24% (42 patients). The AL group had higher values for both NT-proBNP (P=0.0001) and 6-month mortality (P=0.0001). By multivariate analysis, independent predictors of 6-month mortality were higher NT-proBNP (Q4), NYHA class (III-IV), lower cardiac output (<4 L.min-1), and pericardial effusion.

## Conclusions

NYHA, NT-proBNP, cardiac output, and pericardial effusion were independent predictors of mortality in cardiac disease due to any of the three amyloidosis types. NT-proBNP values were highest in AL amyloidosis.

